# Using Next-Generation Sequencing to Detect Differential Expression Genes in *Bradysia odoriphaga* after Exposure to Insecticides

**DOI:** 10.3390/ijms18112445

**Published:** 2017-11-17

**Authors:** Haoliang Chen, Lulu Lin, Farman Ali, Minghui Xie, Guangling Zhang, Weihua Su

**Affiliations:** 1 Institute of Plant Protection and Agro-Products Safety, Anhui Academy of Agricultural Sciences, Hefei 230031, China; chenhaoliang2004@163.com (H.C.); linle2013@163.com (L.L.); drfarman@gmail.com (F.A.); xieminghui730@163.com (M.X.); zhglnxy@126.com (G.Z.); 2 Department of Agriculture, Abdul Wali Khan University, Mardan 23200, Khyber Pakhtunkhwa, Pakistan

**Keywords:** next-generation sequencing, *Bradysia odoriphaga*, differential expression genes, insecticide

## Abstract

*Bradysia odoriphaga* (Diptera: Sciaridae) is the most important pest of Chinese chive. Insecticides are used widely and frequently to control *B*. *odoriphaga* in China. However, the performance of the insecticides chlorpyrifos and clothianidin in controlling the Chinese chive maggot is quite different. Using next generation sequencing technology, different expression unigenes (DEUs) in *B. odoriphaga* were detected after treatment with chlorpyrifos and clothianidin for 6 and 48 h in comparison with control. The number of DEUs ranged between 703 and 1161 after insecticide treatment. In these DEUs, 370–863 unigenes can be classified into 41–46 categories of gene ontology (GO), and 354–658 DEUs can be mapped into 987–1623 Kyoto Encyclopedia of Genes and Genomes (KEGG) pathways. The expressions of DEUs related to insecticide-metabolism-related genes were analyzed. The cytochrome P450-like unigene group was the largest group in DEUs. Most glutathione S-transferase-like unigenes were down-regulated and most sodium channel-like unigenes were up-regulated after insecticide treatment. Finally, 14 insecticide-metabolism-related unigenes were chosen to confirm the relative expression in each treatment by quantitative Real Time Polymerase Chain Reaction (qRT-PCR). The results of qRT-PCR and RNA Sequencing (RNA-Seq) are fairly well-established. Our results demonstrate that a next-generation sequencing tool facilitates the identification of insecticide-metabolism-related genes and the illustration of the insecticide mechanisms of chlorpyrifos and clothianidin.

## 1. Introduction

Chinese chive, *Allium tuberosum* Rottler ex Sprengel (Asparagales: Amaryllidaceae) is considered to be an important vegetable in north China, because it is the main stuffing for dumplings. In the production of Chinese chive, leek maggot, *Bradysia odoriphaga* Yang and Zhang (Diptera: Sciaridae), has always been a major impediment [1]. While the adult of *B. odoriphaga* looks like mosquito, the larva of *B. odoriphaga* is a maggot that feeds on the chive roots and stems, causing plants to stunt or even die. Under favorable temperatures, a female can lay about one hundred eggs on the chive stems or in the moist soil near the chive plants [2]. In north China, *B. odoriphaga* can cause heavy losses to the production of Chinese chive or even ruin the whole crop if not treated with insecticides [3]. Therefore, many studies have been conducted on the ecology and chemical control of *B. odoriphaga* [1,2,3]. Apart from chemicals, some other potential management techniques have also been used [4,5]. Chlorpyrifos, which we tested, has been widely used in Anhui province to control the Chinese chive maggot, whereas clothianidin has not been used against *B. odoriphaga* before. The 48 h LC50 of Chlorpyrifos is as high as 25.1 µg/L, while that of clothianidin is merely 0.5 µg/L. The widespread frequent application of chemical insecticides (e.g., organophosphates, and neonicotinoids) is the most prevalent management strategy used against *B. odoriphaga* when planting the Chinese chive in China. This has led to an excessive use of insecticides resulting in environmental pollution and high residues on marketed chives.

The change of detoxification gene expression plays a key role in insecticide metabolism [6]. Identifying the specific genes involved in insecticide metabolism and their genetic pathways could be beneficial for controlling *B. odoriphaga*. However, little research has been conducted on the insecticide metabolism of this pest; therefore, limited genomic information on this pest is available in GenBank databases [7,8]. Next generation sequencing (NGS) technologies have dramatically developed in the last decade, which allows us to sequence DNA and RNA much more quickly and cheaply than before, and as such has revolutionized the study of genomics and molecular biology [9,10,11]. RNA-Seq has been used to find insecticide-metabolism-related genes in several species, such as *Cimex lectularius* [12], *Plutella xylostella* [13], *Panonychus ulmi* [14] and *Tetranychus cinnabarinus* [15].

In this study, in order to determine insecticide resistant gene/s, we performed RNA-seq for *B. odoriphaga* treated with an organophosphate insecticide, chlorpyrifos, and a neonicotinoid insecticide, clothianidin, with the former being used commonly in the field to control *B. odoriphaga*. The differential expression unigenes (DEUs) were identified using Basic Local Alignment Search Tool (BLAST) and annotated against the National Center for Biotechnology Information (NCBI) Non-Redundant (NR) dataset. Thereafter, the DEUs were categorized by gene ontology (GO) and assigned to pathways using the Kyoto Encyclopedia of Genes and Genomes (KEGG). Finally, 14 unigenes that included five cytochrome P450 (P450)-like unigenes, five glutathione S-transferase (GST)-like unigenes, two carboxylesterase-like unigenes, and two cuticle-protein-like unigenes were used in qRT-PCR to confirm their expression under different insecticide treatments.

Determination of the genetic pathways and specific genes involved in the detoxification pathway could be beneficial for the control of *B. odoriphaga*. This will give us a choice to use alternative methods for controlling this particular pest if it develops resistance to a single specific or group of insecticides.

## 2. Results

### 2.1. RNA-Seq Sequencing and Assembly

De novo transcriptome sequencing of all stages of *B. odoriphaga* generated 34,154 unigenes, with a total length of 38,551,589 bp, a mean length of 1128 bp and an N50 (50% of the total assembled sequence was contained in contigs of this length or longer) of 1984 bp. A total of 20,058 unigenes were annotated against the NCBI NR protein database, 5280 in GO function categories, and 15,910 unigenes were mapped onto the canonical pathways in KEGG. Fifteen samples treated with insecticides on average generated 23.99 Mb raw sequencing reads, of which 23.97 Mb clean reads were obtained for each sample after filtering low quality reads. For all insecticide-treated samples, the percentage of clean reads was higher than 99.10%, with an average of 99.89% (Table 1). There were about 1.20 billion clean nucleotides for each insecticide-treated sample. The Q20 (error probability of 0.01) of the clean nucleotides of all samples was more than 95.50%, with an average of 98.01%. The GC (guanine and cytosine) percentages for each insecticide-treated sample were between 38.76% and 41.53%, averaging 40.42%. The percentage of clean reads from insecticide-treated samples mapped to the reference sequence ranged between 80.97% and 86.17%, with an average of 83.56% (Table 1).

### 2.2. Unigene Expression Analysis for Insecticide Treatment

**Figure 1:**
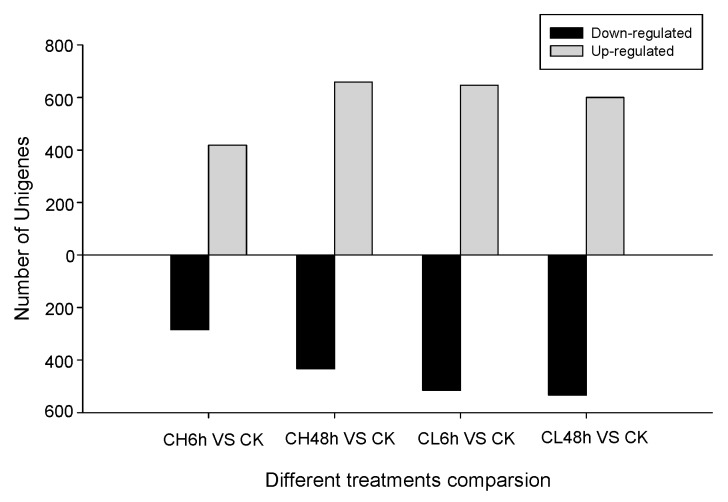
The number of up- and down-regulated unigenes is more than two-fold when compared between different treatments. CK: Control; CH: Chlorpyrifos; CL: Clothianidin; 6 and 48 h: treatment with Chlorpyrifos or Clothianidin after 6 or 48 h.

**Figure 2:**
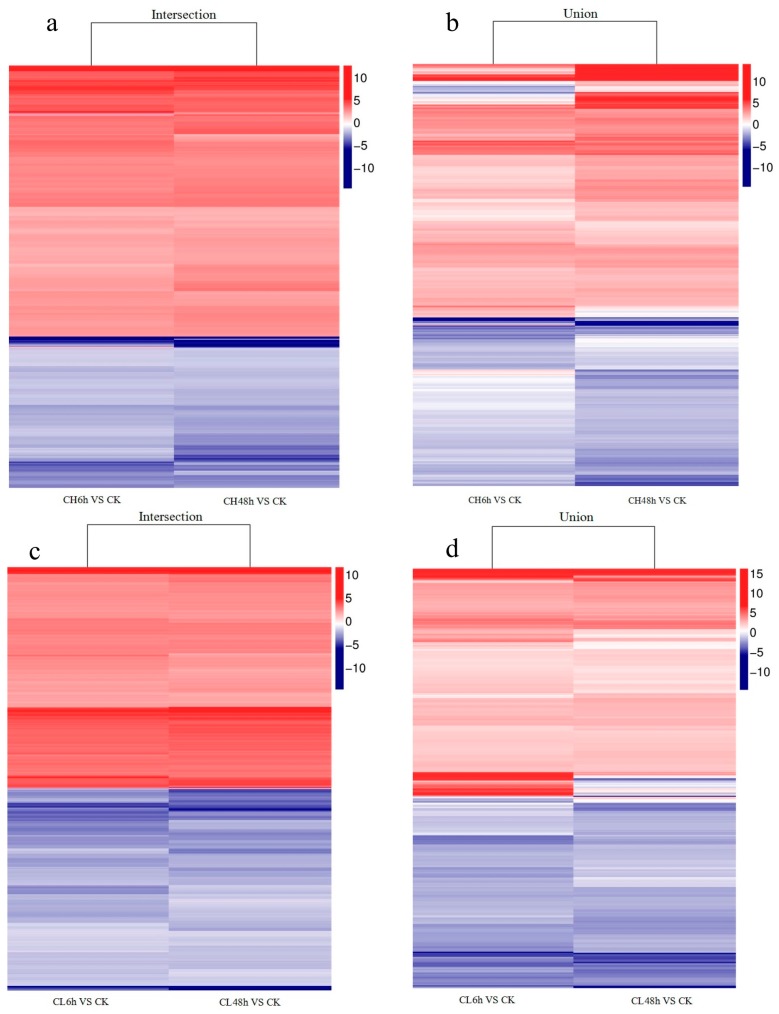
Heat map with dendogram for the Differential Expression Unigenes (DEUs) after treating the larvae with the same insecticide for different time durations. Gradient color barcode at the right indicates fold change of gene expression. CK: Control; CH: Chlorpyrifos; CL: Clothianidin; 6 h and 48 h: treatment with Chlorpyrifos or Clothianidin after 6 or 48 h. (**a**,**b**): DEUs for chlorpyrifos treated for 6 and 48 h in comparison with control, (**a**) is intersection and (**b**) is union; (**c**,**d**): DEUs for clothianidin treated for 6 and 48 h in comparison with control, (**c**) is intersection and (**d**) is union.

**Figure 3:**
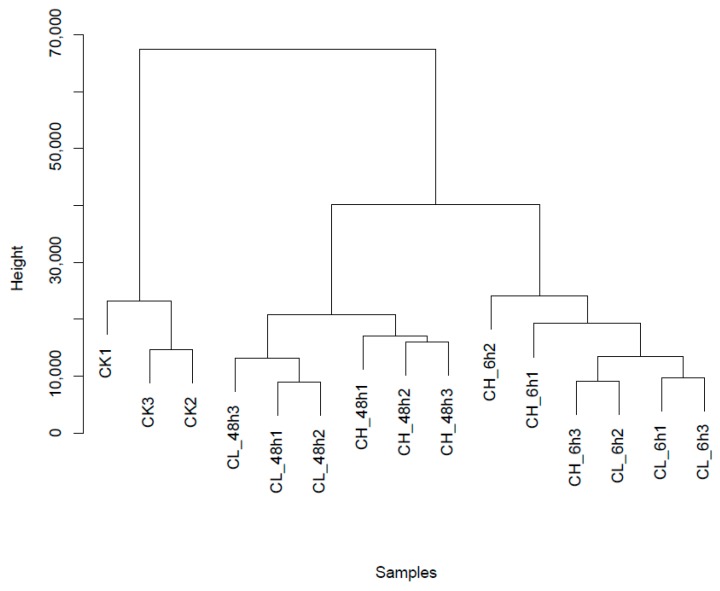
Cluster tree involving all samples. The distances of the expressed gene are calculated by the Euclidean method. Meanwhile, the algorithm of the sum of squares of deviations is used to calculate the distance between samples so that a cluster tree can be built. The Y axis means the height in the cluster tree. When samples have similar height values, they can be easily gathered. CK: Control; CH: Chlorpyrifos; CL: Clothianidin; 6 and 48 h: treatment with Chlorpyrifos or Clothianidin after 6 or 48 h.

Clean reads were mapped to the assembled reference sequence database of *B. odoriphaga*. The number of identified unigenes was between 29,146 and 31,467, with an average of 30,736 for all samples, and the proportion to de novo database was between 85.34% and 92.13%, with an average of 89.99% (Table S1). The unigene expression of samples treated with insecticides, was compared with control based on the fragments per kilobase of transcript per million mapped reads (FPKM) value. A total of 703 to 1161, with an average of 1022 unigenes were considered as expression-changed unigenes after pesticide treatment (Figure 1). *B. odoriphaga* exposed to chlorpyrifos for 48 h had the maximum number (658) of up-regulated unigenes in comparison to the control, followed by 6 h exposure to clothianidin (646), while the maximum number (533) of down-regulated unigenes was observed in the treatment exposed to clothianidin for 48 h and the minimum number (284) was found in the treatment exposed to chlorpyrifos for 6 h (Figure 1). The intersection and union of the DEU heat map for the same insecticide treatment and different time duration is shown in Figure 2. The intersection of the DEU number for chlorpyrifos and clothianidin treated samples with different time durations is 371 and 681, respectively, while for union it is 1414 and 1600, respectively (Spreadsheet S1). The cluster tree indicates that the clustering of insecticide-treated samples are clearly separated from the control. Within insecticide treated samples, the insects exposed to insecticides for 6 h formed a separate cluster to those exposed for 48 h, although there were two different insecticides used (Figure 3).

### 2.3. GO Classifications and KEGG Pathway Identification of DEUs

**Figure 4:**
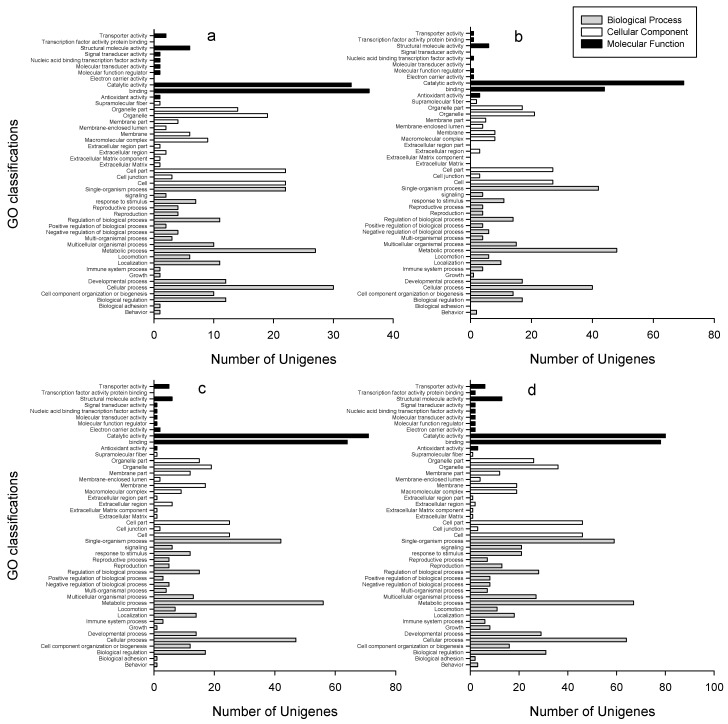
Number of DEUs classified into the Gene Ontology (GO) function catalogue using different insecticides and different time durations in comparison with control. (**a**) treatment with Chlorpyrifos for 6 h versus control, (**b**) treatment with Chlorpyrifos for 48 h versus control, (**c**) treatment with Clothianidin for 6 h versus control, (**d**) treatment with Clothianidin for 48 h versus control.

The identified DEUs were assigned to GO functional classification. For the DEUs, the samples treated with different insecticides for different time durations when compared with the control, generated a total of 370–863 unigenes, with an average of 581 that can be classified into 41–46 categories of the GO functional group (Figure 4). The DEUs of clothianidin treated for 48 h in comparison with control had the most number of unigenes that can be classified into the GO terms, of which 863 unigenes were assigned to 46 GO classifications. In contrast, chlorpyrifos-treated samples for 6 h versus control had the least classification unigenes, of which 370 unigenes were assigned into 44 GO classifications (Table S2). For the DEUs, samples treated with two different insecticides for different time durations when compared with each other, generated a total of 110–225 unigenes, with an average of 149 that could be assigned into 24–35 categories of GO functional groups (Table S2). The stability of the percentages of DEUs for the three main GO categories was noted in the samples treated with insecticides versus control, rather than in insecticide versus insecticide-treated samples (Table S3). For example, the percentage of DEUs assigned to the biological process was in the range of 48.92 to 52.61 for insecticide-treated samples in comparison with control, but within insecticide-treated samples, the percentage ranged from 38.46 to 51.56 (Table S3).

**Figure 5:**
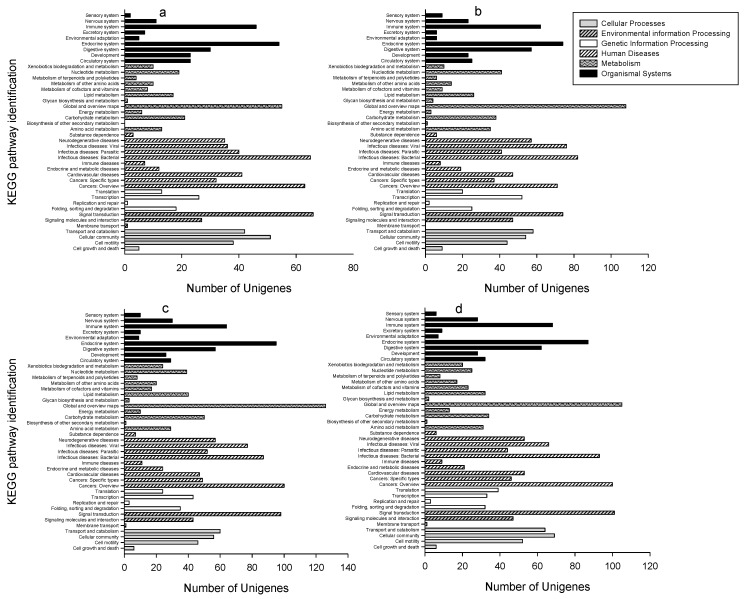
Number of DEUs assigned to KEGG pathway enrichment using different insecticides and different time durations in comparison with control. (**a**) treatment with chlorpyrifos for 6 h versus control, (**b**) treatment with chlorpyrifos for 48 h versus control, (**c**) treatment with clothianidin for 6 h versus control, (**d**) treatment with clothianidin for 48 h versus control.

To identify possible active biological pathways of DEUs, the unigenes were mapped onto the canonical pathways in KEGG. For the DEUs in the insecticide-treated sample versus control and using different exposure times, 354 to 658 DEUs, with an average of 544, could be mapped into 987 to 1623 pathways, with an average of 1399 (Figure 5). The DEUs for clothianidin-treated samples for 6 h in comparison with control can be mapped onto most pathways (1623), while chlorpyrifos treated samples for 6 h could be mapped onto the least (987) (Table S4). For the DEUs, after comparing the insecticide-treated samples with each other for different exposure times, a total of 178 to 354 DEUs, with an average of 215, could be mapped onto 496–735 pathways, with an average of 589. The majority of the pathways (735) identified by the DEUs to be mapped after comparing the insecticide-treated samples for different time durations, were achieved with chlorpyrifos-treated samples for 6 and 48 h, while the least number (496) was achieved with clothianidin-treated samples after 6 and 48 h exposure (Table S4). The ten most up- and down-regulated DEUs in the control samples and those treated with two pesticides for 48 h are listed in Table 2 and Table 3. Fifteen out of the twenty most significant DEUs from the control and chlorpyrifos-treated samples can be mapped onto KEGG pathways, while this was eighteen out of twenty for clothianidin.

### 2.4. Insecticide-Metabolism-Related DEUs and Quantitative Real-Time PCR Validation

**Figure 6:**
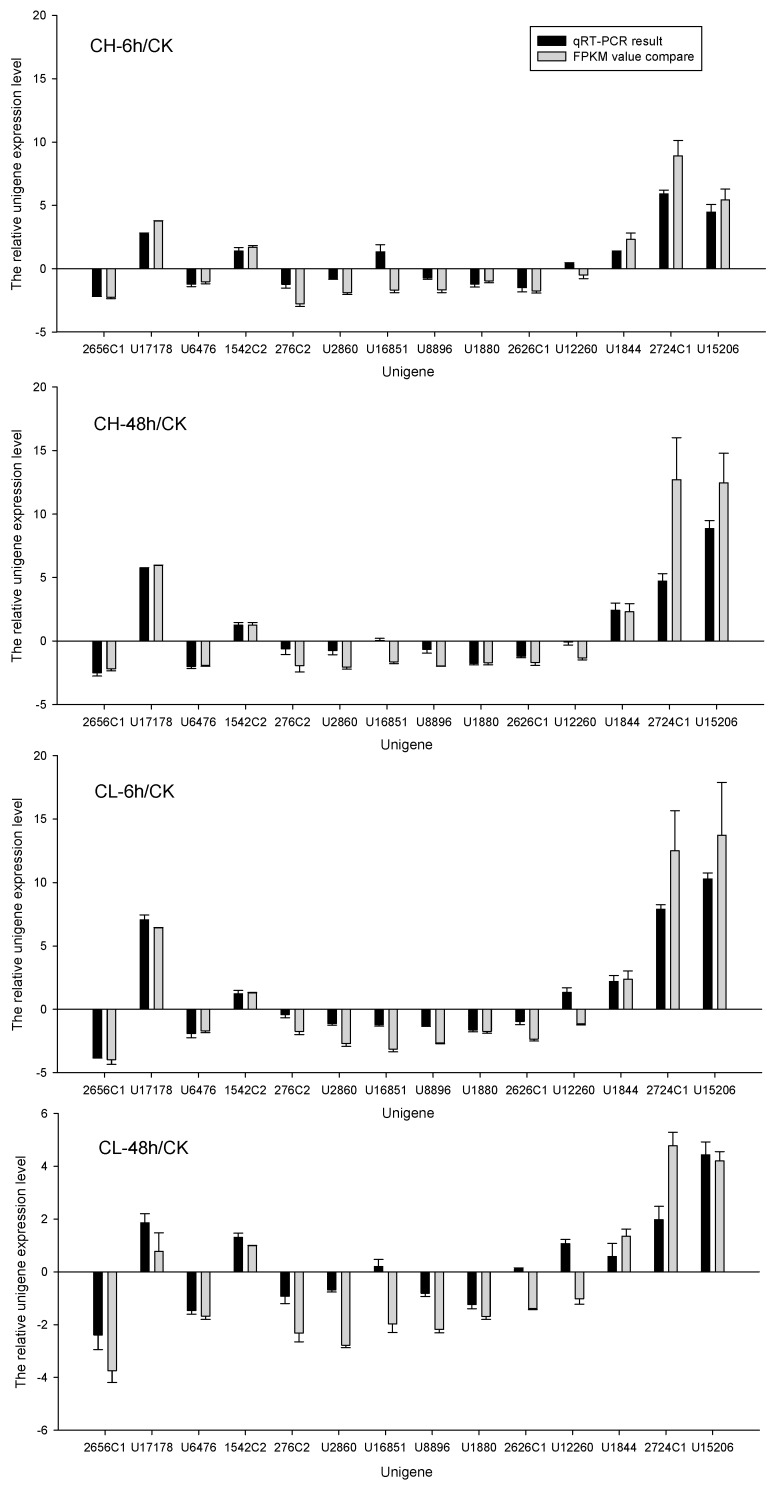
Insecticide-metabolism-related unigenes in comparison with control as confirmed by Quantitative Real-Time Polymerase Chain Reaction (qRT-PCR) and the calculated fragments per kilobase of transcript per million mapped reads (FPKM) values. CK: Control; CH: Chlorpyrifos; CL: Clothianidin; 6 h: treated 6 h; 48 h: treated 48 h. In the relative gene expression level (log2R) on *Y*-axis, R indicates the ratio of unigene expression in insecticides treated as compared with that in control.

An analysis of the expression changes of insecticide-metabolism-related unigenes, such as carboxylesterase [16], catalase [17], cytochrome P450 [18], GST [19], nicotinamide adenine dinucleotide (NADH) dehydrogenase [20], trypsin [21], superoxide dismutase [22], acetylcholinesterase [23], gamma-aminobutyric acid (GABA) receptor [24], nicotinic acetylcholine receptor [25], and sodium channel [26,27] after insecticide treatment is given in Table 4. More than 50 P450-like unigenes were found to be expression-changed in all insecticide-treated samples in comparison with control, and the group of DEUs related to the P450-like unigenes was the largest group of DEUs related to insecticide metabolism (Table 4). Most GST-like unigenes were down-regulated after insecticide treatment, while only the same one GST-like unigene was up-regulated in the chlorpyrifos-treated sample for 6 and 48 h, and the clothianidin-treated sample for 6 h. For clothianidin-treated samples for 48 h, all DEUs related to GST were down-regulated. The DEUs related to the GABA receptor showed consistency after insecticide treatment where all the three DEUs related to the GABA receptor were the same and were down-regulated. The same four DEUs related to the nicotinic acetylcholine receptor were found after treating the larvae with either chlorpyrifos or clothianidin for 6 or 48 h; two of which were up-regulated and two down-regulated. Same eight DEUs related to the sodium channel were up-regulated when treated with chlorpyrifos for 6 and 48 h, whereas in the case of clothianidin-treated larvae for 6 and 48 h, only one out of eight DEUs related to the sodium channel was down-regulated, with seven up-regulated. Fourteen unigenes, including five P450-like unigenes, five GST-like unigenes, two carboxylesterase-like unigenes, and two cuticle-protein-like unigenes were used for the qRT-PCR to confirm the relative expression in different treatments. Figure 6 shows the results after comparing the qRT-PCR and FPKM value. Most of the chosen unigenes showed consistency of expression, however, seven out of 56 contrasts did not agree well.

## 3. Discussion

Understanding of the unigenes involved in insect detoxification can provide new ideas for pest management, but insecticide metabolism is a complex biological event and is related to insecticide target proteins, and other cellular processes [28]. Next-generation sequencing technologies can promote research into insecticide detoxification in insects with unknown genomes or incomplete protein databases and help us to obtain more comprehensive knowledge of insecticide detoxification mechanisms. The underlying mechanism that involves the inhibition of acetylcholinesterase (AChE) is considered the major molecular mechanism of organophosphates against insects [29], whereas neonicotinoids kill the insects by continuous stimulation of nervous system through nicotinic acetylcholine receptors (nAChRs). Previous studies have mainly focused on these two mechanisms and the genes related to them. One concern that needs to be addressed, however, is to find out other gene/s that could be involved in the process of insect killing by chlorpyrifos and clothianidin. In this study, we found many DEUs after analyzing insecticide-treated samples versus control. Those DEUs were assigned to GO functional classification and KEGG pathway analysis.

For the Illumina HiSeq platform, the clean reads ranged between 90% and 99%, and clean nucleotides Q20 usually around 97.0% [7,30,31]. In this study, the average of clean reads and clean nucleotides, Q20, was 99.89% and 98.01% respectively. These data show that BGISEQ-500 is a reliable platform for next generation sequencing. The clean reads mapped to the library sequence in the present study (80.97% to 86.17%, with an average of 83.56% for 15 samples) was a little higher than that of Xie [32], which ranged from 78.35% to 82.18%, and lower than that of Ma [30], which ranged from 90.46% to 93.25%, but almost the same as that of the Gao [8], who used the same species obtaining clean reads ranging between 79.91% and 85.76%.

In the current study, the number of DEUs related to insecticide metabolism using the same insecticide but for different durations, was not much different, which shows that most insecticide-metabolism-related unigenes respond in 6 h (Figure 2). The DEUs of up-regulated and down-regulated patterns is almost the same for the intersection when treated with the same insecticide for different time durations (Figure 2a,c). However, for union, there were still some unigenes whose expression was not uniform (Figure 2b,d). The results of the cluster tree showed that the samples treated for either 6 or 48 h were clustered separately. This may indicate that the treatment duration of insecticide has more impact on the expression of unigenes than the type of insecticide (Figure 3).

The number of DEUs could be assigned to a GO functional classification and a GO functional group number, both of which were much smaller in the insecticide versus insecticide-treated samples, than in insecticide versus control. The same situation was noted for the DEUs mapped onto the KEGG pathways (Tables S2 and S4). The percentages of DEUs assigned to the three main GO categories were more stable in the insecticide versus control samples than in the insecticide versus insecticide samples (Table S3). This may indicate that the presence or absence of insecticide is a major factor for the DEUs of the insecticide-treated versus control treatment, which could be assigned into GO categories. However, comparing the insecticide-treated samples to each other does not seem to have much impact. Nevertheless, the duration of the insect exposure to these insecticides may determine their assignment to GO categories. In the significant DEUs of 48 h exposure to chlorpyrifos vs. control treatment, two of the 10 most up-regulated DEUs were annotated as a cuticle protein (Table 2). Cuticle proteins are associated with the regulation of molting and metamorphosis in insects [33]. In mosquitoes, insecticide resistance is also attributed to the chitinized cuticle, because it may impede the absorbance of the insecticide [34,35]. The cuticle protein genes differentially express as deltamethrin-susceptible and deltamethrin-resistant strains of *Culex pipiens pallens* [36], and are considered to have a role for resistance to deltamethrin in *C. pipiens pallens* [37]. Our results indicate that cuticle proteins may have a role in the detoxification of pesticides as well. In the most down-regulated unigenes for chlorpyrifos and clothianidin, both have one unigene mapped into the KEGG pathway, numbered K00011 and K07604 (Table 2 and Table 3). In the present study, Lysosomal protein-like unigene (CL1023.Contig2) was listed in the 10 most up-regulated DEUs after 48 h exposure to chlorpyrifos. This protein has proved to be involved in the resistance of *Musca domestica* to organophosphorus insecticides [38]. Heat shock proteins (HSPs) are a family of proteins that are produced in response to stressful conditions, and their expression changes under the stress of insecticides [39]. Our results also show one HSP67 in the most down-regulated DEUs after 48 h exposure to clothianidin. Two unigenes mapped onto K00011 were annotated as aldo-keto reductase protein and aldose reductase protein in the NCBI Nucleotide database (Table 2 and Table 3). Reductase protein plays an important role in insecticide detoxification metabolism, such as NADPH-Cytochrome P450 Reductase [40,41]. The most significant DEUs showed differences after treatment with clothianidin and chlorpyrifos, which may be due to the different modes of action of these insecticides, possible different impacts on off-target molecules or cellular/physiological pathways, or differences in how these insecticides may be metabolized based on their different structures.

Increasing the activity of the insecticide detoxification enzyme can help insects to survive insecticide exposure. Many enzyme families, such as P450, GST, and carboxylesterases are considered to be involved in insecticide detoxification mechanisms [42]. Cytochrome P450 monooxygenase-mediated metabolism is a common mechanism of insect detoxification to organophosphorus and neonicotinoid insecticides [43]. In each treatment, more than 50 DEUs were related to P450, which is considered the largest group of insecticide-metabolism-related unigenes. Also, it occupies a large proportion of all P450 genes, because insect genomes probably carry about 100 P450 genes [43]. GST is another large group of insecticide-related-unigenes, the number of which in the insect genomes is variable [44]. For example, *Apis mellifera* only contains eight GST genes [45], but *Culex quinquefasciatus* contains 38 GST genes [46]. In our study, each treatment had more than 30 DEUs related to GST, suggesting that almost all GSTs responded to insecticide treatment. Esterases, which include a large group of enzymes and which are considered as a mediated metabolic resistance of insecticides, have been detected in almost all insects [17]. The number of DEUs related to carboxylesterase in the chlorpyrifos-treated samples for 48 h, was greater than those treated with chlorpyrifos for 6 h, which may indicate that carboxylesterase-related unigenes respond more slowly than other insect-metabolism-related unigenes. This may provide an explanation as to why in some insects (brown plant hopper, mosquitoes, and aphids) the amplification of gene copies of carboxylesterase are shown in resistant strains, because more gene copies could help compensate for a slow transcriptional response [47,48,49]. Also, in some insects (mosquitoes, flies), mutations in carboxylesterase genes occur, showing enhanced detoxification of insecticide, perhaps to also compensate for the slow transcription of this family of detoxification proteins [50,51,52]. Most NADH dehydrogenase-related DEUs were down-regulated in each treatment, but Zhang et al. [53] report that the NADH dehydrogenase subunit six was up-regulated after the Allyl isothiocyanate fumigation, regardless of the concentration or fumigation time. This may be caused by the different insecticides used or different methods of insecticide application. In the current study, however, we fed the chive stem treated with insecticide to the insect larva, whereas Zhang et al. used fumigation. NADH dehydrogenase is the largest respiratory complex and the first enzyme of the mitochondrial electron transport chain. NADH dehydrogenase may behave differently and can have a different response mechanism to the way insecticides are applied, such as fumigation or feeding. In this study, we found many DEUs in *B. odoriphaga* after treating them with chlorpyrifos and clothianidin for 6 and 48 h. Analysis of these DEUs can expand our understanding of resistance and detoxification mechanisms. Also, in future study, the DEUs can be treated as target genes for the detoxification mechanisms of *B. odoriphaga*. The specific genes involved in detoxification could be considered as molecular targets to explore novel approaches to control *B. odoriphaga* and to save the production of Chinese chive.

## 4. Materials and Methods

### 4.1. Insect Rearing and Insecticide Treatment

The initial culture of Chinese chive maggot (*Bradysia odoriphaga*) was collected from the bulbs of Chinese chive in Bozhou, Anhui province, China (115°53′46.68″ E, 33°59′45.6″ N) in March 2016. The maggot was then maintained in the laboratory on the stem of Chinese chives. In order to keep the Petri dish moistened, 1.5% agarose gel was heated and poured into the Petri dish to make a layer at the bottom of Petri dish. After the gel was solidified, a filter paper was placed on top of the gel. Chive stem was cut into 1–2 cm pieces and transferred to a plastic bowl with a cover on top. Several pairs of adults were then introduced into the bowl using an aspirator and covered with a lid. The adults laid eggs on the chive stems, which were then transferred to the Petri dish prepared before. After 7–10 days, the newly hatched larvae were shifted onto a fresh food. The food was refreshed after every 3–4 days until the larvae pupated. The rearing conditions were controlled by a walk-in climate chamber maintained at 25 ± 1 °C, a photoperiod of 14 h light: 10 h darkness and 70 ± 10% relative humidity [54]. Eggs, second instar larvae, fourth instar larvae, pupae and adults were collected for de novo transcriptome sequencing as a reference database. One milliliter insecticide solutions of chlorpyrifos (widely used for the control of Chinese chive maggots in Anhui province) and clothianidin (has not been used against chive maggot before in Anhui province) @ 25.1 and 0.5 µg/L respectively, (concentration was based on the LC50 value calculated after 48 h exposure) were added onto filter paper in the Petri dishes, which were used for rearing the fourth instar larvae of *B. odoriphaga*. The chive stems were plunged into the insecticide solution for 5 s, and then fed to the fourth instar larvae for either 6 or 48 h. Fourth instar larvae of *B. odoriphaga* feeding on chive stem (plunged in water for 5 s) were considered as a control. Each treatment was replicated three times and the live larvae post-treatment were collected for RNA extraction and sequencing.

### 4.2. RNA Extraction and cDNA Library Preparation

Total RNA was extracted according to the manufacturer’s protocol of an RNA isolation system (SV Total RNA Isolation System Kit, Promega Corporation, Madison, WI, USA). From the extracted RNA samples, the possible residual genomic DNA was removed by treating it with deoxyribonuclease (Dnase I: Fermentas Inc., Burlington, ON, Canada). RNA quality was determined using Agilent 2100 (Agilent Technologies, Santa Clara, CA, USA). The RIN (RNA integrated number) values of the sample chosen for RNA-Seq were between 6.4 and 7.2 (Table S5). The chosen RNA samples were treated as per standard procedure. Briefly, Oligo (dT) magnetic beads were used to select mRNA with a polyA tail and the target RNA obtained after purification. The target RNA was fragmented and reverse-transcribed to double-stranded cDNA (dscDNA) by N6 random primer. The dscDNA fragments were first end repaired with phosphate at 5′ end and stickiness “A” at 3′ end, then adapters were ligated to the dscDNA fragments. PCR was performed to amplify the ligation product. The PCR product was then denatured by heat and the single strand DNA was cyclized by splint oligo and DNA ligase. Finally, the prepared library was sequenced by BGISEQ-500 sequencing platform.

### 4.3. RNA-Seq Sequencing and DEU Screening

Since no genomic sequence in any database was available for *B. odoriphaga*, we used a unigene database of *B. odoriphaga* transcriptome for all stages as a library sequence. The reference unigenes database was sequenced by Illumina HiSeq 2500 platform (Illumina, San Diego, CA, USA). The raw reads were deposited in the NCBI Sequence Read Archive database under the accession number SRP107066. The de novo assembly, sequence clustering, and functional categorization were done using the same method as that of Chen et al. [7]. Insecticide-treated samples were sequenced by BGISEQ-500 sequencing platform at the Beijing Genomics Institute (BGI; Shenzhen, China) and the raw reads were deposited in the NCBI Sequence Read Archive database under the accession number SRR5512549-SRR5512563. Clean reads were obtained after removing the reads with adaptors, reads having unknown nucleotides more than 5%, and reads having more than 50% bases with Q-value ≤ 20. The software Bowtie2 was used to map the clean reads to assembled reference sequence database and then the fragment for each unigene was counted from the mapping results [55]. The relative expression of the unigenes was calculated by dividing the unigene’s FPKM value in insecticide-treated samples with the same unigene FPKM value in control. We then identified the DEUs with an absolute value of log_2_Ratio > 1 or log_2_Ratio < −1. The formula for FPKM value is as follows:

Where C is the number of fragments that is uniquely aligned to the calculated unigene, N is the total number of fragments that is uniquely aligned to all unigenes and L is the base number of the calculated unigene.

### 4.4. DEUs, GO Annotation and Pathway Enrichment among Different Treatments

GO is an international standard in functional classification systems. Blast2go PRO software was used for analyzing DEUs assigned to a GO functional group [56], and unigenes with annotation were distributed into three main ontologies: molecular function, cellular component, and biological process [57]. Pathway analysis helps to further understand the DEUs’ biological functions. The detected DEUs were also annotated against the Kyoto Encyclopedia of Genes and Genomes (KEGG) database [58].

### 4.5. Sequence Confirmation and Quantitative Real-Time Polymerase Chain Reaction (qRT-PCR) Validation

Thirteen expression-changed unigene sequences including five CytochromesP450 (P450) genes, five glutathione S-transferase (GST) genes, two AChE genes, and one reference gene glyceraldehyde-3-phosphate dehydrogenase (GAPDH), were confirmed by qRT-PCR. Specific primers were designed using Primer premier 5.0, and then reverse transcription PCR was conducted. After electrophoresis of the PCR products, the DNA band was extracted by Agarose Gel Extraction kit (Takara Biotechnology (Dalian) Co., Ltd., Dalian, China), and then subcloned into vector (pEASY-T1 Simple Cloning Kit, Beijing TransGen Biotech Co., Ltd., Beijing, China) according to the manufacturer’s protocol. A 3730 DNA Analyzer was used to determine the nucleotide sequences. The confirmed sequences were deposited in the GenBank database with the accession numbers KY997061-KY997073, and MG407793-MG407796.

The relative expression of 14 confirmed sequences was determined by qRT-PCR, choosing the GAPDH as the reference gene. Beacon Designer 7.0 was used to design the primers for qRT-PCR, and one cycle amplification efficiency of the primers was calculated [59]. cDNA was synthesized from one microgram of total RNA after removing the genomic DNA (PrimeScript™ RT Reagent Kit with gDNA Eraser, Takara Biotechnology (Dalian) Co., Ltd.). Cycle threshold (*C*t) value of GAPDH in each cDNA sample was used to adjust the concentrations of cDNA [60]. The qRT-PCR was performed on a CFX96 Real-Time PCR Detection System (Bio-Rad Laboratories, Inc., Hercules, CA, USA) using SYBR Premix (Takara Biotechnology (Dalian) Co., Ltd.) according to the manufacturer’s instructions. The thermal cycling conditions were: 45 cycles at 95 °C for 30 s, 56 °C for 20 s, and 72 °C for 30 s. There were four biological samples and each had two technical replicates to determine the relative expression of unigenes. The delta *C*t value of the target gene and reference gene for each sample was first calculated, and then the relative expression to the reference gene was determined by using the delta *C*t value and the efficiency of the primer. The relative expression of each gene in pesticide-treated samples vs. control was calculated. The NCBI accession number, primers designed for confirmation and qRT-PCR are listed in Table S6. The PCR amplification efficiencies of primers for qRT-PCR are shown in Table S7.

## 5. Conclusions

This study provides information about the genes of *B. odoriphaga* involved in responding to two insecticides, chlorpyrifos and clothianidin. This information can expand our understanding of resistance and detoxification mechanisms of this insect. The study further provides analysis of the most significantly up- and down-regulated DEUs and insecticide metabolism related DEUs. These genes could be further studied and used as molecular targets to explore novel approach to control *B. odoriphaga*. 

## Figures and Tables

**Table 1:** Statistics of clean reads.

Sample	Clean Reads	Clean Reads (%)	Clean Nucleotides (bp)	>Q20 (%)	GC (%)	Total Mapped Reads (%)
CK1	24,129,440	99.96	1.21 × 10^9^	98.18	40.70	88.36
CK2	24,122,549	99.94	1.21 × 10^9^	98.28	39.85	83.93
CK3	24,117,751	99.91	1.21 × 10^9^	98.34	38.76	83.85
CH_6h1	23,946,892	99.95	1.20 × 10^9^	95.52	40.35	80.97
CH_6h2	23,953,030	99.98	1.20 × 10^9^	98.43	41.06	86.17
CH_6h3	23,940,066	99.92	1.20 × 10^9^	98.21	40.62	83.63
CH_48h1	23,949,969	99.96	1.20 × 10^9^	98.37	40.20	83.07
CH_48h2	23,741,893	99.10	1.19 × 10^9^	98.18	40.79	84.57
CH_48h3	23,944,154	99.94	1.20 × 10^9^	98.35	41.53	84.96
CL_6h1	23,945,882	99.95	1.20 × 10^9^	98.13	40.16	80.98
CL_6h2	23,949,511	99.96	1.20 × 10^9^	98.22	40.29	83.80
CL_6h3	23,948,469	99.96	1.20 × 10^9^	98.01	40.31	81.25
CL_48h1	23,919,748	99.84	1.20 × 10^9^	98.30	40.62	83.34
CL_48h2	23,949,005	99.96	1.20 × 10^9^	97.89	40.33	81.82
CL_48h3	23,945,861	99.95	1.20 × 10^9^	98.43	40.67	82.78

CK: Control; CH: Chlorpyrifos; CL: Clothianidin; 6 and 48 h: treatment with Chlorpyrifos or Clothianidin after 6 or 48 h.

**Table 2:** Most significant DEUs in control samples vs. those exposed to chlorpyrifos for 48 h.

Unigene ID	Annotation	Expression Compared to Control	Fold Change	KEGG Annotation
CL2310.Contig3	malate synthase A	Up	14.06	K01638
CL546.Contig2	glycine-rich cell wall structural protein	Up	14.06	K10385
Unigene21344	spliceosome-associated protein	Up	13.96	K13806
CL2019.Contig2	keratin, type II cytoskeletal	Up	13.37	K11422
Unigene14906	gastrointestinal growth factor	Up	12.78	-
CL2724.Contig1	cuticle protein	Up	12.70	K03006
Unigene15206	cuticle protein	Up	12.46	K03006
CL1023.Contig2	Lysosomal protein	Up	12.13	-
Unigene11830	glycine-rich cell wall structural protein	Up	11.97	K13098
CL2019.Contig1	keratin, type II cytoskeletal	Up	11.92	K07605
Unigene22580	acyl-CoA desaturase	Down	−6.93	K00507
Unigene21624	endocuticle structural glycoprotein	Down	−8.62	-
Unigene343	cell target antigen	Down	−9.15	-
CL534.Contig2	activating signal cointegrator	Down	−9.17	K13114
CL1382.Contig3	adhesion G-protein coupled receptor	Down	−9.44	-
Unigene1839	Full=Flexible cuticle protein	Down	−9.92	-
CL2888.Contig2	aldo−keto reductase protein	Down	−10.00	K00011
CL572.Contig1	BTB/POZ domain-containing protein	Down	−10.32	K10457
CL347.Contig2	Succinyl-CoA ligase (ADP/GDP-forming) subunit α, mitochondrial	Down	−10.63	-
Unigene6098	Zonadhesin	Down	−14.62	K07604

**Table 3:** Most significant DEUs in control samples vs. those exposed to clothianidin for 48 h.

Unigene ID	Annotation	Expression Compared to Control	Fold Change	KEGG Annotation
CL2903.Contig2	Titin	Up	11.58	K14721
Unigene14493	zinc finger protein	Up	10.16	K09228
CL884.Contig2	Mediator of RNA polymerase	Up	10.12	K15169
CL2635.Contig1	serine/threonine-protein kinase	Up	9.78	K08791
Unigene3337	amino acid adenylation domain-containing protein	Up	9.72	K00142
Unigene5847	tyrosine-protein phosphatase	Up	9.52	K18040
CL234.Contig1	AT-rich interactive domain-containing protein	Up	9.46	K07874
CL565.Contig3	transcription factor	Up	9.38	K09269
CL2809.Contig2	transcription initiation factor	Up	9.38	K14650
Unigene2261	histone acetyltransferase	Up	9.25	K11306
CL2708.Contig1	aldose reductase	Down	−7.81	K00011
Unigene26463	dienelactone hydrolase family protein	Down	−8.96	K01061
Unigene21654	heat shock protein 67	Down	−9.12	K01011
Unigene20053	keratin, type I cytoskeletal	Down	−9.49	K07604
Unigene456	serine protease inhibitor	Down	−9.65	-
Unigene15229	RNA recognition motif domain containing protein	Down	−9.92	K13195
Unigene13983	larval serum protein	Down	−10.46	K00505
Unigene21431	transmembrane protein	Down	−10.54	-
CL119.Contig1	cofilin/actin-depolymerizing factor	Down	−13.77	K05765
CL2528.Contig3	cytochrome P450	Down	−14.62	K14999

**Table 4:** DEUs related to insecticide metabolism. Up: Up-regulated expression; Down: Down-regulated expression. Up- or down-regulation indicates that the unigene’s FPKM value for insecticide-treated samples is two times more or 0.5 times less than that in control.

Gene Name	CH6h VS. CK		CH48h VS. CK		CL6h VS. CK		CL48h VS. CK	
	Up	Down	Up	Down	Up	Down	Up	Down
*Cytochrome P450*	30	23	35	18	35	35	21	40
*Glutathione S-transferase*	1	31	1	29	1	37	0	35
*Carboxylesterase*	1	2	7	14	4	16	2	13
*Acetylcholinesterase*	5	2	5	6	7	5	4	6
*NADH dehydrogenase*	1	11	2	7	2	13	2	11
*Catalase*	3	1	4	1	2	1	3	1
*Trypsin*	10	14	20	34	18	18	8	38
*Superoxide dismutase*	3	2	1	1	1	4	2	0
*GABA receptor*	0	3	0	3	0	3	0	3
*Nicotinic acetylcholine receptor*	2	2	2	2	2	2	0	3
*Sodium channel*	8	0	8	0	7	1	7	1

CK: Control; CH: Chlorpyrifos; CL: Clothianidin; 6 h: treated 6 h; 48 h: treated 48 h.

## Supplementary Materials

Supplementary materials can be found at www.mdpi.com/1422-0067/18/11/2445/s1.
