# Supplementary material for: Using Next-Generation Sequencing to Detect Differential Expression Genes in Bradysia odoriphaga after Exposure to Insecticides
Source: Int J Mol Sci. 2017 Nov 17;18(11):2445. doi: 10.3390/ijms18112445 (PMC5713412; doi:10.3390/ijms18112445)
Supplement: Supplementary file 1 [file ijms-18-02445-s001.zip › ijms-236023-supplementary/ijms-236023-supplementary.pdf]

Table S1. The number of identified unigenes in *de novo* database and their proportion

Sample name	Identified expressed unigenes	Proportion in *de novo* database (%)
CK1	29146	85.34
CK2	31163	91.24
CK3	30282	88.66
CH_6h1	31087	91.02
CH_6h2	30350	88.86
CH_6h3	30746	90.02
CH_48h1	30785	90.14
CH_48h2	30840	90.30
CH_48h3	31022	90.83
CL_6h1	31355	91.80
CL_6h2	30301	88.72
CL_6h3	31467	92.13
CL_48h1	30895	90.46
CL_48h2	31092	91.03
CL_48h3	30509	89.33

CK: control; CH_6: Chlorpyrifos treated 6 hours; CH_48h: Chlorpyrifos treated 48 hours; CL_6h: Clothianidin treated 6 hours; CL_48h: Clothianidin treated 48 h.

Table S2: GO categories of DEUs.

	CH6h VS CK	CH48h VS CK	CL6h VS CK	CL48h VS CK
Behavior	1	2	1	3
Biological adhesion	1	0	1	2
Biological regulation	12	17	17	31
Cell component organization or biogenesis	10	14	12	16
Cellular process	30	40	47	64
Developmental process	12	17	14	29
Growth	1	1	1	8
Immune system process	1	4	3	6
Localization	11	10	14	18
Locomotion	6	6	7	11
Metabolic process	27	48	56	67
Multicellular organismal process	10	15	13	27
Multi-organismal process	3	4	4	7
Negative regulation of biological process	4	6	5	8
Positive regulation of biological process	2	4	3	8
Regulation of biological process	11	14	15	28
Reproduction	4	4	5	13
Reproductive process	4	4	5	7
response to stimulus	7	11	12	21
signaling	2	4	6	21
Single-organism process	22	42	42	59
Cell	22	27	25	46
Cell junction	3	3	2	3
Cell part	22	27	25	46
Extracellular Matrix	1	0	1	1

Extracellular Matrix component	1	0	1	1
Extracellular region	2	3	6	2
Extracellular region part	1	0	1	1
Macromolecular complex	9	8	9	19
Membrane	6	8	17	19
Membrane-enclosed lumen	2	4	2	4
Membrane part	4	5	12	12
Organelle	19	21	19	36
Organelle part	14	17	15	26
Supramolecular fiber	1	2	1	1
Antioxidant activity	1	3	1	3
binding	36	44	64	78
Catalytic activity	33	70	71	80
Electron carrier activity	0	1	2	2
Molecular function regulator	1	1	1	2
Molecular transducer activity	1	0	1	2
Nucleic acid binding transcription factor activity	1	1	1	2
Signal transducer activity	1	0	1	2
Structural molecule activity	6	6	6	13
Transcription factor activity protein binding	0	1	0	2
Transporter activity	2	1	5	6
total	370	520	572	863

Table S3. DEUs' percentage assigned into three main GO categories.

**Main Categories**	**CH6h VS CK**	**CH48h VS CK**	**CL6h VS CK**	**CL48h VS CK**	**CL6h VS CH6h**	**CL48h VS CH48h**	**CH48h VS CH6h**	**CL48h VS CL6h**
biological process	48.92	51.35	49.48	52.61	40.91	41.18	38.46	51.56
cellular component	28.92	24.04	23.78	25.14	22.73	32.77	24.48	24.00
molecular function	22.16	24.62	26.75	22.25	36.36	26.05	37.06	24.44

CK: Control; CH: Chlorpyrifos; CL: Clothianidin; 6h: treated 6 h; 48h: treated 48 h.

Table S4. KEGG pathway identification of DEUs

**Pathway Identification**	**CH6h VS CK**	**CH48h VS CK**	**CL6h VS CK**	**CL48h VS CK**
Cell growth and death	5	9	6	6
Cell motility	38	44	46	52
Cellular community	51	54	56	69
Transport and catabolism	42	58	60	64
Membrane transport	1	0	1	1
Signaling molecules and interaction	27	47	43	47
Signal transduction	66	74	98	101
Folding, sorting and degradation	18	25	35	32
Replication and repair	1	2	3	3
Transcription	26	52	43	33

Translation	13	20	24	39
Cancers: Overview	63	71	100	100
Cancers: Specific types	32	37	49	46
Cardiovascular diseases	41	47	47	53
Endocrine and metabolic diseases	12	19	24	21
Immune diseases	7	8	11	9
Infectious diseases: Bacterial	65	82	87	93
Infectious diseases: Parasitic	40	41	52	44
Infectious diseases: Viral	36	76	77	66
Neurodegenerative diseases	35	57	57	53
Substance dependence	3	6	7	6
Amino acid metabolism	13	35	29	31
Biosynthesis of other secondary metabolism	0	1	1	1
Carbohydrate metabolism	21	38	50	34
Energy metabolism	6	3	10	13
Global and overview maps	55	108	126	105
Glycan biosynthesis and metabolism	1	4	3	2
Lipid metabolism	17	26	40	32
Metabolism of cofactors and vitamins	8	9	17	23
Metabolism of other amino acids	10	14	20	17
Metabolism of terpenoids and polyketides	4	6	8	8
Nucleotide metabolism	19	41	39	25
Xenobiotics biodegradation and metabolism	10	10	24	20
Circulatory system	23	25	29	32
Development	23	23	26	28
Digestive system	30	57	57	62
Endocrine system	54	74	95	87
Environmental adaptation	5	6	9	7
Excretory system	7	6	10	9
Immune system	46	62	64	68
Nervous system	11	23	30	28
Sensory system	2	9	10	6
Total	987	1409	1623	1576

Table S5. RNA-Seq samples' value detected by Agilent 2100.

Sample name	concentration (ng/μL)	volume (μL)	total amount (μg)	RNA Integrated Number
egg	579	60	34.7	7.2
2nd instar larva	714	300	214.2	7.2
4th instar larva	1056	200	211.2	7.1
pupa	1096	80	87.7	6.9
adult	624	120	74.9	7.2
CK-1	562	60	33.72	6.4
CK-2	428	80	34.24	6.7
CK-3	476	60	28.56	6.5
CH_6h-1	832	80	66.56	6.9
CH_6h-2	732	80	58.56	7.1

CH_6h-3	525	160	84.00	7.0
CH_48h-1	600	60	36.00	7.0
CH_48h-2	573	80	45.84	7.0
CH_48h-3	657	60	39.42	7.0
CL_6h-1	450	160	72.00	7.0
CL_6h-2	744	80	59.52	7.0
CL_6h-3	603	160	96.48	7.0
CL_48h-1	748	60	44.88	6.9
CL_48h-2	708	60	42.48	6.9
CL_48h-3	868	40	34.72	6.9

CK: Control; CH: Chlorpyrifos; CL: Clothianidin; 6h and 48h: treatment with Chlorpyrifos or Clothianidin after 6 or 48 h.

Table S6. The primers to confirm unigenes and RT-qPCR.

	Gene name	Unigene number	accession number in NCBI		PCR product size (bp)
Primers to comfirm unigenes	P450	CL2656.Contig1	KY997061	F: ATACTTCTTGGCGTTATTTTCAAAT<br>R: CATGGGTCTCCTATTCAGTGATAC	1554
		Unigene17178	KY997062	F: TGGATTGCGTTCGAAACATT<br>R: AATTCACGTAATTCGGCACG	2098
		Unigene 6476	KY997063	F: TTCGCTCAAAGGTGTGTTACAA<br>R: ATATGACGTGTTGCAAAAGATGA	682
		CL1542.Contig2	KY997064	F: CTGCTGTAAAGGTTGATCTGTTATC<br>R: TCAGACGAGGTTAAGATGAGTGC	1450
		CL276.Contig2	KY997065	F: CGGATTATCTGACCTCAAATACG<br>R: GAAATGTGGCGAATCGCATA	357
	GST	Unigene2860	KY997066	F: TTTGATTTACTGTAAAAAGGAGAACC<br>R: AATGTTATCCGACGTTAGTATTC	898
		Unigene16851	KY997067	F: CATTTTTTCCCACACCATTGT<br>R: TTGGTTTCTATCTATTGAACGTGAA	764
		Unigene8896	KY997068	F: TGTAATGCAAAATCTACGCACAG<br>R: TTAGTATTCGTAGCGGTCGCA	846
		Unigene1880	KY997069	F: ACAGATACACGATCAGTTATCGTGA<br>R: TCGTTGCTACTAATCAGTTGAACC	1899
		CL2626.Contig1	KY997070	F: TTATGTGCTCTACGTGTTTCTGAAT<br>R: CATACGTAGCAGTTACGAGTTCG	1593
	Carboxylesterases	Unigene12260	MG407793	F: TCTAAAAGATCAAGTTGAAGCTTTG<br>R: GCAAAATTGTAGTACATATCGACG	927
		Unigene1844	MG407794	F: GTTGCAAGGCACGCTTATAAT<br>R: TCGATGAAATAGTCCAGCCG	974
	Cuticle protein	CL2724.Contig1	MG407795	F: TAAGGAAATAAAATCTTTTTACGCA<br>R: TACAACTGTGAATATGCAATTGTTG	718
		Unigene15206	MG407796	F: AAAATGGCATTCAAGTTTTTGAC<br>R: GTTTTCAAACAGTTTGTTCAAAGAC	693
	GAPDH	Unigene19803	KY997073	F: TTATCTTCTCAGAAATTTGCCCTT<br>R: TATTCTATAACACGGTTTTAATGCC	1425
Primers for RT-qPCR	P450	CL2656.Contig1	KY997061	F: ATATGGCTGTTCCCTTTTCC<br>R: CAACTGTCCTATGATGCTGTTAGT	160
		Unigene17178	KY997062	F: TTGCTCCAGCTTTTCATCAG<br>R: AACACTTTGCCCACCTCTTT	106
		Unigene 6476	KY997063	F: TGTTCGCTCAAAGGTGTGTT<br>R: TTCGTTGTTAAATTGGCAGG	146
		CL1542.Contig2	KY997064	F: AACAGACGGATGCAATCGA<br>R: CAAGACAAGGCAAGGGAAG	126
		CL276.Contig2	KY997065	F: GTCATCATACCTGTCTACTCGCT<br>R: GGAAGGAATACACTGCTGTCTTT	131
	GST	Unigene2860	KY997066	F: TCATCCAGATAATCGGCAATA<br>R: CATACGCTCAGCGAACACAC	196
		Unigene16851	KY997067	F: TACTCGGAGACTTCGGGTAGA<br>R: AATTCGGAGGCAATAATCATG	157
		Unigene8896	KY997068	F: TTCATCCAGATAATCGGCAA<br>R: CATACGCTCAGCGAACACA	197
		Unigene1880	KY997069	F: ATTGAGACTGCTTCTCTTCTGC<br>R: GGTTTTGTTTTTTTATGTTCGG	106
		CL2626.Contig1	KY997070	F: GGTTACCAGACGTCCAGACAC<br>R: GACCCACCTACGACAATCAAT	159
	Carboxylesterases	Unigene12260	MG407793	F: GCTGGTGGTAGTAGTGTTCATTT	130

			R: GAGCTTTTTCTCTTGGTTTTTG	
	Unigene1844	MG407794	F: CTTAAGTTCGTTTTGCGGTC	124
			R: TAGTCTCTTCCATGCTTTGGTT	
Cuticle protein	CL2724.Contig1	MG407795	F: GCCCTTGTTTCTTTTATTGCT	200
			R: GATCGGGTTTCCTGTTGATT	
	Unigene15206	MG407796	F: AAGACGGTTTGACGGGTG	184
			R: GCTGCTACGATTTTTGCG	
GAPDH	Unigene19803	KY997073	F: CGAAATCGTTGAAGGTCTGA<br>R: CTGGAATGATGTTTTGCTGG	122

F: forward; R: reverse; PCR: polymerase chain reaction; RT-qPCR: reverse transcription quantitative. Comfirm unigenes PCR primer Tm were set 58°C and RT-qPCR primer were set 56°C. GAPDH was considered as reference gene.

Table S7. The amplification efficiencies of primers for qRT-PCR

Gene name	**Unigene number**	**Curve fitting linear**	R^2	**amplification efficiencies**
P450	CL2656.Contig1	Y=3.33x+16.80	0.99	1.99
	Unigene17178	Y=3.25x+21.42	0.99	2.05
	Unigene 6476	Y=3.32x+17.95	0.99	1.99
	CL1542.Contig2	Y=3.40x+13.24	0.99	1.96
	CL276.Contig2	Y=3.46x+17.45	0.99	1.95
GST	Unigene2860	Y=3.44x+14.40	0.99	1.95
	Unigene16851	Y=3.36x+15.48	0.99	1.98
	Unigene8896	Y=3.46x+14.37	0.99	1.95
	Unigene1880	Y=3.50x+16.52	0.99	1.93
	CL2626.Contig1	Y=3.50x+14.06	0.99	1.93
Carboxylesterases	Unigene12260	Y=3.59x+18.11	0.99	1.90
	Unigene1844	Y=3.35x+19.94	0.99	1.99
Cuticle protein	CL2724.Contig1	Y=3.57x+18.54	0.99	1.91
	Unigene15206	Y=3.48x+17.35	0.99	1.94
GAPDH	Unigene19803	Y=3.41x+12.03	0.99	1.96
